# JTIS: enhancing biomedical document-level relation extraction through joint training with intermediate steps

**DOI:** 10.1093/database/baae125

**Published:** 2024-12-19

**Authors:** Jiru Li, Dinghao Pan, Zhihao Yang, Yuanyuan Sun, Hongfei Lin, Jian Wang

**Affiliations:** School of Computer Science and Technology, Dalian University of Technology, No. 2 Linggong Road, Ganjingzi District, Dalian 116024, China; School of Computer Science and Technology, Dalian University of Technology, No. 2 Linggong Road, Ganjingzi District, Dalian 116024, China; School of Computer Science and Technology, Dalian University of Technology, No. 2 Linggong Road, Ganjingzi District, Dalian 116024, China; School of Computer Science and Technology, Dalian University of Technology, No. 2 Linggong Road, Ganjingzi District, Dalian 116024, China; School of Computer Science and Technology, Dalian University of Technology, No. 2 Linggong Road, Ganjingzi District, Dalian 116024, China; School of Computer Science and Technology, Dalian University of Technology, No. 2 Linggong Road, Ganjingzi District, Dalian 116024, China

## Abstract

Biomedical Relation Extraction (RE) is central to Biomedical Natural Language Processing and is crucial for various downstream applications. Existing RE challenges in the field of biology have primarily focused on intra-sentential analysis. However, with the rapid increase in the volume of literature and the complexity of relationships between biomedical entities, it often becomes necessary to consider multiple sentences to fully extract the relationship between a pair of entities. Current methods often fail to fully capture the complex semantic structures of information in documents, thereby affecting extraction accuracy. Therefore, unlike traditional RE methods that rely on sentence-level analysis and heuristic rules, our method focuses on extracting entity relationships from biomedical literature titles and abstracts and classifying relations that are novel findings. In our method, a multitask training approach is employed for fine-tuning a Pre-trained Language Model in the field of biology. Based on a broad spectrum of carefully designed tasks, our multitask method not only extracts relations of better quality due to more effective supervision but also achieves a more accurate classification of whether the entity pairs are novel findings. Moreover, by applying a model ensemble method, we further enhance our model’s performance. The extensive experiments demonstrate that our method achieves significant performance improvements, i.e. surpassing the existing baseline by 3.94% in RE and 3.27% in Triplet Novel Typing in F1 score on BioRED, confirming its effectiveness in handling complex biomedical literature RE tasks.

**Database URL**: https://codalab.lisn.upsaclay.fr/competitions/13377#learn_the_details-dataset

## Introduction

The development of Biomedical Natural Language Processing (BioNLP) has made it possible to automatically mine important information from medical literature, encompassing interactions between genes and proteins [1–3], drug reactions in cancer [4], and chemically induced diseases [5]. However, biomedical Relation Extraction (RE) remains a complex component within the biomedical information retrieval pipeline. It plays a crucial role in analyzing biomedical concepts and their relationships in textual data, which significantly drives the development of drug discovery and personalized medicine. In the field of biology, most existing RE tasks focus on sentences that involve single pairs of entities and their relationships [6–10]. For example, the Drug–Drug Interaction (DDI) corpus [4] annotates relationships only when two drugs appear in the same sentence. However, real-world relationships often occur between different types of entities, and describing a complete biological process or relationship often requires multiple sentences, leading current research to shift from sentence-level to document-level analysis [11]. Document-level (DocRE) targets the entire document [12, 13], and given the complexity of entity information distributed across multiple sentences and the semantic and discursive structure of documents, models must be able to accurately handle entities scattered across several sentences and infer relationships through the integration of multiple pieces of information [14]. Additionally, in the field of biology, discovering new relationships between entities, such as those between a drug itself or between a disease and a drug, is fundamental for asserting relationships. Medical research during the COVID-19 pandemic highlights the urgent global need for treatment and vaccine solutions to alleviate human suffering. In this context, the research community faces unprecedented challenges in identifying newly emerging biomedical entities. Therefore, developing automated methods that can work reliably in unknown environments and accurately identify complex relationships has become crucial.

Previous research has typically focused on specific types of biomedical relationships, such as chemical–disease relationships (CDR) and gene–disease associations (GDA) [15]. Although existing models perform well on these datasets, DocRE technologies have not yet fully addressed the challenges of interactions among multiple types of biological entities in complex scenarios. In contrast, the BioRED dataset [16] offers a more complex DocRE environment, involving interactions among multiple entity types such as gene, disease, chemical, and cell line. This not only demands higher performance from models but also emphasizes the importance of developing high-performance models capable of recognizing complex biomedical entity interactions to support further development of downstream applications such as knowledge graph construction and medical information retrieval.

**Figure 1.**
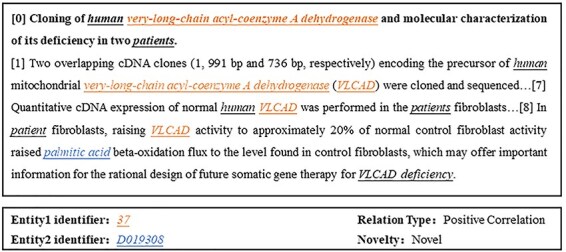
Example document and a relation triple from BioRED, where “37” and “D019308” are unique identifiers corresponding to the color-marked mentions in the text, with Entity 1 and Entity 2 highlighted in colorfully underlined italics and other entities in black underlined italics.

In the field of biology, describing the interactions between two biological entities often requires multiple sentences, and the complexity of these relationships makes DocRE a challenge [17]. In context, an entity typically has various mentions. Figure 1 provides an example from BioRED where “very-long-chain acyl-coenzyme A dehydrogenase” and “VLCAD” appear in different sentences but represent the same entity. Ignoring cross-sentence entity interactions and coreference information, merely extracting these relationships from a single sentence would be insufficient. Existing customized biomedical models such as BioBERT [18] and PubMedBERT [19] have overlooked the importance of cross-sentence entity interactions, particularly the role of coreference information, resulting in performance deficiencies.

From the texts of the BioRED dataset, we automatically extract various biomedical entities such as drugs and diseases, along with their associated relationships, and determine whether they represent novel relationships in the text. The image in Fig. 1 presents a data entry from the BioRED dataset, consisting of a title and abstract from biomedical literature. These data are annotated with mentions of all biomedical entities contained within the literature, with single or multiple mentions corresponding to the same entity, each marked with a unique identifier. The types of relationships between pairs of entities are also annotated, without directional indications, and are classified into relationships. The entity pairs that are novel findings are marked as “Novel”; otherwise, if they are previously known background knowledge, they are marked as “No.”

**Figure 2.**
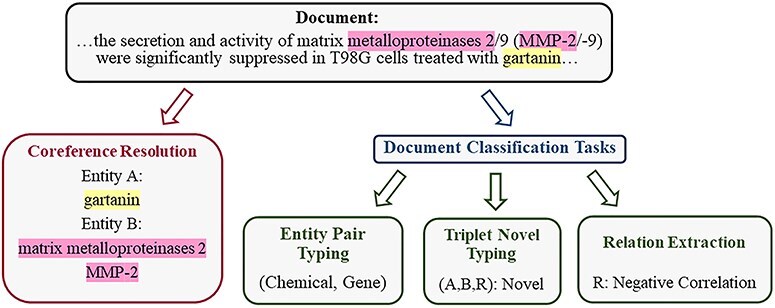
The example, adapted from BioRED, illustrates the division of the training task into four related tasks, from an input document with annotated entity mentions to RE and TNT output.

In the field of biology, most previous methods implicitly learn entity and contextual information, which leads to ineffective supervision. Therefore, we propose a multitask learning method that enhances the intermediate steps of RE to improve the performance of the language model in the field of biology. This approach is designed to enhance the model’s capabilities in related tasks, thereby improving its ability to capture contextual information and entity type information. It is tailored specifically for the complexities and demands of the biomedical literature, aiming to extract higher-quality relationships. Consider the motivating example in Fig. 2:

(1) Coreference Resolution (CR): The descriptions “matrix metalloproteinases 2” and “MMP-2” in the document refer to the same entity in the gene database [20], representing different mentions of the same entity within the text. Modeling coreference relationships is crucial for the model to capture entity semantics contextually. Therefore, for a given document, we use CR to parse various contextual representations conveyed by different mentions of the same entity.

(2) Entity Pair Typing (EPT): Entity pair types play a significant guiding role in type determination. In the BioRED dataset, distinct entity pair types correspond to different sets of relation types. We construct entity pair types as labels used during training by extracting manually annotated entity type combinations from data for each sample. Within pairs of entities, their type information can be used to filter out improbable relationships.

By incorporating these two additional designed tasks, we have constructed a multitask model capable of handling four tasks: CR, EPT, RE, and TNT. Through multitask tuning, the model demonstrates significant performance improvements in both RE and TNT tasks. These four intermediate steps are interconnected. CR, RE, and TNT capture the textual context, while EPT maintains information about the type of entity pairs. We explicitly supervise the model on the outputs of these intermediate steps through carefully designed tasks.

In summary, our main contributions are as follows: (i) We propose the JTIS method, which involves jointly training on the CR, EPT, RE, and TNT tasks. This approach enhances the intermediate steps of RE, alleviates the problem of error accumulation, and improves the performance of relation classification and end-to-end joint RE. (ii) Our proposed adversarial training and model ensemble methods further enhance the model’s performance and robustness in both tasks. (iii) Experimental results demonstrate that our method is effective and outperforms previous state-of-the-art methods on the BioRED dataset leaderboard by 3.94% in RE and 3.27% in TNT in F1 score.

## Related work

In the field of biology, most existing datasets typically focus on direct relationships between two entities within a single sentence. For instance, the DDI corpus only annotates interactions when two drugs appear in the same sentence. However, describing a complete biological process or relationship often requires multiple sentences, thus driving the need for DocRE datasets. Widely used datasets, like the CDR and GDA datasets, include the CDR dataset, which contains 1500 PubMed abstracts centered on binary interactions between chemicals and diseases, and the GDA dataset which emphasizes associations between genes and diseases. Although expanding the scope, these datasets still focus primarily on binary relationships.

To address the complexity and diversity of relationships in the biomedical field, the recently introduced BioRED dataset presents a more advanced challenge. It covers six types of biomedical entities—gene, disease, chemical, variant, species, and cell line, and eight types of biologically meaningful non-directional relationships, such as association, positive correlation, and negative correlation, also annotating the novelty of these relationships. BioRED comprises 600 manually annotated PubMed articles and includes an additional 400 titles and abstracts annotated for the Biocreative competition, incorporating research related to COVID-19, making it a more challenging and interactive DocRE dataset.

Existing DocRE methods focus on using heterogeneous document Graph Neural Networks [21] or Pre-trained Language Models (PLM) [22] to implicitly learn context and entity type encoding. Widely used DocRE models in the biomedical field, such as BERT-GT [23] and PubMedBERT, are based on the Transformer architecture. However, these methods typically use sequences of words as input and may inadequately capture complex interactions between entities, particularly in handling complex relationships that require inference [24]. When entities span sentences or even paragraphs, especially when they are far apart in the document, extracting relationships becomes increasingly difficult, and an increase in coreference phenomena in the text further complicates finding the correct relationships. Xu *et al*. [25] are the first to incorporate entity structural information into the model by identifying coreference mentions and their relationships both within and outside sentences, significantly improving the modeling of entity interactions. Peng *et al*.’s studies [26–28] emphasize the importance of effectively utilizing textual context and entity type information, proposing enhanced training strategies to explicitly guide models in learning these complexities. The effective integration of entity types and coreference information helps in building more precise RE models.

## Methods

**Figure 3.**
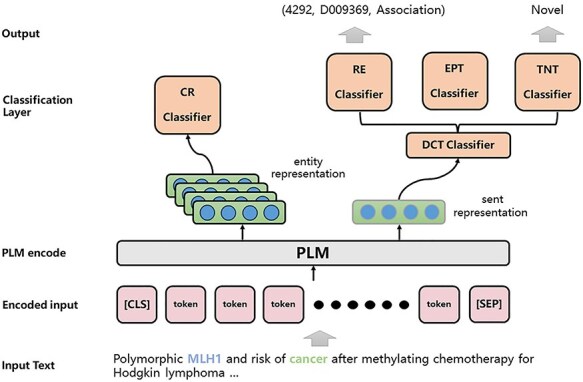
The overall architecture of our proposed JTIS model.

The architecture of the JTIS model is shown in Fig. 3. In the following sections, we detail the data preprocessing, multitask approach, model training, and model ensemble.

### Problem definition

Consider a document $D$ containing sentences ${S_d} = {s_i}_{i = 1}^{\left| {{S_d}} \right|}$, entities ${E_D} = {e_i}_{i = 1}^{\left| {{E_D}} \right|}$, and tokens ${T_D} = {t_i}_{i = 1}^{\left| {{T_D}} \right|}$. In this document, each entity $e \in {\ }{E_D}$ is assigned an entity type $c \in C$ and has one or more mentions ${M_e} = {m_i}_{i = 1}^{\left| {{M_e}} \right|}$. For a pair of entities $({e_i},{\ }{e_j})$, the goal of document-level RE is to predict whether there exists a certain type of relationship between them, based on whether the mentions of ${e_h}$ and ${e_t}$ express a certain relationship $r \in R$. Here, $C$ and $R$ are predefined sets of entity and relationship types, and “None” is also considered as a category, used to indicate the absence of a relationship. Additionally, for each pair of entities with a valid relationship $r \in R$, it is necessary to determine if their relationship is a novel relation $N \cup \left\{ {NA} \right\}$ in this context, where $\left\{ {NA} \right\}$ indicates that the relationship of the entity pair is not a novel relation discovered in this document, and $N$ represents the opposite meaning.

### Preprocess

We utilize a domain-specific PLM called PubMedBERT for context encoding in the field of biology. PubMedBERT is developed specifically for biomedical texts and generates its vocabulary from scratch. It incorporates biomedical domain knowledge into the BERT-based pre-training, achieving state-of-the-art performance on various biomedical natural language understanding tasks.

In more detail, for each document $D$, we inserted “[CLS]” at the beginning and “[SEP]” at the end of the text. For every entity pair $({e_i},{\ }{e_j})$ within the document, we construct a sample $s$, regardless of whether they have a relationship. For each mention $m \in {M_D}$ in the sample, we insert special symbols “${\mathrm{@\backslash E}}$“and “${\mathrm{E/@}}$“to identify the positions of mentions. During the training process, we filter out samples that involve entity pair types for which no candidate relationships exist. As shown in equation (1), we tokenize the document, composed of titles and abstracts, and input it at the token level into the PLM to obtain encoded representations of the text.


(1) $$V = PLM\left( {{x_1},{x_2}, \ldots ,{x_z}} \right)$$

Where $V \in {^{\left\{ {m \times h} \right\}}}$ is the encoded representation of preprocessed input text. $z$ is the maximum length of the input text. $h$ is the embedding dimension of the $PLM$.

### Multitask method

#### Coreference resolution

We define the relationships between mentions within the BioRED dataset based on whether they refer to the same standardized entity. We categorize the relation of mentions as coreference if the mentions refer to the same standardized entity. Our objective is to equip a pre-trained encoder with the capability to automatically identify whether mention pairs are coreference using this newly defined task. We use the representation of the special symbol “${\mathrm{@\backslash E}}$“in $V$ as the representation for mention $m$. Consider a pair of mentions $({m_i},{m_j})$, we use a bilinear layer to compute the probability of the CR task:


(2) $$P_{i,j}^{CR} = \delta \left( {{m_i}{A_{CR}}{m_j} + {\ }b} \right)$$

Where $\delta $ is the softmax function, ${A_{CR}} \in {^{\left( {h \times 2 \times h} \right)}}$ is a trainable neural layer, which attends to the mentions simultaneously and $b \in $ is the model’s prior bias. As shown in equation (3), we employ cross-entropy to calculate the loss for the CR task, which is used to update the parameters of the $CR$ classifier and the encoding layer in the model.


(3) $$L_s^{CR} = CrossEntropy\left( {P_{i,j}^{CR},\bar y_{i,j}^{CR}} \right)$$

Where $\bar y_{i,j}^{CR}$ is the coreference label extracted from the annotation data.

#### Document classification tasks

Within BioRED, there are inherent constraints and mapping relationships between entity types and relationship categories. Therefore, we believe that enhancing the entity type recognition capability of the model can significantly enhance the quality of relationship category recognition. To address this, we have established the EPT task based on annotated data. In essence, it involves extracting manually labeled entity type combinations for each sample from existing data to construct entity pair types as labels for training.

In contrast to individually classifying entities, this approach provides a more cohesive task structure and allows for a broader focus on holistic information during classification. As we create a sample for each distinct entity pair within the same document, and all mentions of two different entities in a sample are enclosed by special symbols, we define EPT, TNT, and RE tasks as a unified document classification format. It is worth mentioning that during training, labels of triples with relationships are directly used for training. This means that the calculation of TNT only considers samples with relationships. Samples without relationships do not pass through the TNT classification layer. Specifically, for a sample constructed for a pair of entities $({e_i},{\ }{e_j})$, we ignore the concept of head and tail entities based on the definition of the dataset. We create separate classifiers for different tasks ${t_i} \in \left\{ {EPT,RE,TNT} \right\}$, and utilize the “[CLS]” representation $v_{i,j}^{\left[ {cls} \right]}$ with three distinct classifiers to obtain predictions for these three tasks:


(4) $$P_{i,j}^{{t_i}} = {\delta }\left( {{W_{{t_i}}}v_{i,j}^{\left[ {cls} \right]} + {b_{{t_i}}}} \right)$$

where ${W_{{t_i}}} \in {^{n \times h}}$ is a trainable neural layer, $b \in $ is the model’s prior bias and $n$ is the number of classes in tasks. Similarly, we also use cross-entropy to obtain the loss for DCTs and update the parameters of the classifiers for each task and the shared encoding layer:


(5) $$L_s^{{t_i}} = CrossEntropy\left( {P_{i,j}^{{t_i}},\bar y_{i,j}^{{t_i}}} \right)$$

#### Multitask train

In essence, our objective is to elevate the performance of both the RE task and the TNT task. We achieve this by integrating all tasks through the minimization of a multitask learning objective. We allocate distinct loss weights to all tasks and train the models separately for the RE and TNT tasks. For the RE model, the training loss is expressed in equation (6):


(6) $${L_s} = L_s^{RE} + \sum {\eta ^{{t_i}}}L_d^{{t_i}}$$

During training, all triples with no relationships are also considered as negative examples for the TNT Task. During testing, the RE classifier is used to predict the relationships of triples. Based on the predictions from the RE classifier, triples that are identified as having relationships are then passed through the TNT classifier for novel classification. To enhance the performance and robustness of the single model, we employ an adversarial training approach [29] after loss backpropagation. As shown in equation (7), based on the gradient $g$ at the embedding layer, we add an adversarial perturbation ${r_{adv}}$ to the word vector $x$ to generate adversarial samples. The newly generated adversarial samples are fed into the model to generate the adversarial loss ${L_{adv}}$, and the losses are back-propagated to obtain the gradients generated by the adversarial sample losses at each layer of the model. Since the gradients generated by the original sample losses have not yet been used by the optimizer for parameter updating, the gradients from the original samples are accumulated with those from the adversarial samples. We use this accumulated gradient to update the parameters of the model, effectively improving the performance and robustness of the model.


$${r_{adv}} = \epsilon \\[-12pt]$$

(7) $${x_{adv}} = x + {r_{adv}} \\[-12pt]$$

$${L_{adv}} = Model\left( {{X_{adv}}} \right) \\[-12pt]$$

### Ensemble model

For the final test, we use the k-fold cross-validation method to improve our performance. We integrate the original training set and validation set as a new training set, and use 10-fold cross-validation method to train 10 different weights models, and then integrate the prediction results of each model through voting. Through this method, we use the diversity of the training set to obtain models with different targeted capabilities, thereby improving the RE and TNT performance and robustness of the entire system.

## Experiments

### Datasets

BioRED consists of 600 manually annotated PubMed articles. In our experiment, the dataset is divided into training (500) and validation (100). Additionally, an additional set of 400 annotated PubMed articles is used as test set. Therefore, the dataset used in the experiment consists of 1000 titles and abstracts. Notably, compared to the original dataset in the BioRED paper, what was previously the test set is now used as the validation set, while the combination of the original validation set and training set now serves as the training set. In this dataset, each pair of entities corresponds to only one type of relationship. The statistics of the BioRED dataset, including the numbers of the entities, mentions, and relations in the training and development sets, are presented in Table 1.

**Table 1.** The statistics of the BioRED dataset Novel Relation Pairs mean that pairs describe novel relationships for the context as opposed to background knowledge

Split	#Docs	#Mentions	#Entities	#Relations	#Novel relation pairs
Train	500	16 884	3664	5370	3673
Dev	100	3535	982	1163	859
Test	400	15 400	3597	6034	3683

### Implementation details and evaluation metrics

Our experiments are conducted on an NVIDIA RTX 3090 GPU, and we perform five trials with different seeds to report average values. Our model implementation is based on the PyTorch version of the Hugging Face Transformers library [30], with settings that include two attention heads, two iterations, and a batch size of two. The embedding dimension $H$ of PubMedBERT is 768. The fine-tuning learning rate for BERT is 2e-5, and for other parameters, it is 1e-4. We use the AdamW optimizer with an initial learning rate set to 1e-6, employing a linear warm-up for the first 6% of the training steps, followed by a linear decay of the learning rate to zero. Gradient clipping is applied to model parameters with a maximum norm of 1, and the training duration is set for 100 epochs. All related task weights $\left( {{{\eta }_{Task}}} \right)$ (tasks include CR, TNT, RE, and EPT) are set to 0.1. Performance evaluation is primarily based on the F1 score.

### Main results

We evaluated the performance of the models from three aspects: (i) Entity pair: Assessing whether there is a relationship between the entities without considering the type of relationship; (ii) Entity pair + Relationship type: Once the correct entity pair is identified, evaluating the correct semantic relationship between the two entities; (iii) Entity pair + Novelty: Once the correct pairing and the correct semantic relationship type are identified, assessing whether there is a novelty factor. As shown in Table 2, the results indicate that our single model approach not only achieves better results in the assessment of correct entity pairs and entity pairs + relationship types but also more accurately evaluates the novelty metric for triples, achieving 79.82%, 68.10%, and 60.13% respectively in the test set. This demonstrates that auxiliary tasks built around RE and novelty type steps significantly contribute to the semantic representation and performance enhancement of the model. Additionally, by adopting a model ensemble approach, our model’s performance is further enhanced. This highlights the positive impact of our proposed ensemble method in leveraging model diversity and enhancing the overall system’s robustness.

**Table 2.** RE and TNT results on BioRED with labeled entities (%)

	BioRED dev				BioRED test			
Method	Entity pair	+Relation type		+Novelty	Entity pair		+Relation type	+Novelty
PubMedBERT	78.02		66.14	57.21		74.29	51.93	38.90
JTIS	79.82		68.10	60.13		74.40	55.04	43.01
$JTI{S_{ensemble}}$	81.89		69.66	61.93		75.59	56.67	44.41
Median	–		–	–		73.51	53.12	40.55
Mean	–		–	–		62.34	43.82	32.32

In the absence of provided labeled entities, we utilize PubTator’s API [31] to obtain entities and their normalization results, integrating them with our model to achieve the final experimental outcomes as shown in Table 3. In the two tables above, we also present the median and average results of all participants’ submissions in the BioRED track of the Biocreative challenge. It is evident that our model demonstrates a significant overall performance improvement. However, due to the increase in steps required to complete the task and issues of error accumulation, the model’s performance declines without labeled entities. Specifically, as illustrated in Fig. 1, Entity 1 with identifier “37” may have multiple mentions in the text, such as “very-long-chain acyl-coenzyme A dehydrogenase” and “VLCAD.” When this entity needs to be normalized, it could have a large number of candidate identifiers. As a result, even if the correct mention is identified, it is often challenging to classify it under the correct identifier. If an incorrect entity identifier is extracted, even correctly identified relationship-type triplets cannot be considered correct. In summary, directly using the API approach results in a high number of entity normalization errors, leading to significant cumulative errors.

**Table 3.** RE and TNT results on BioRED without labeled entities (%)

	BioRED dev			BioRED test			
Method	Entity Pair	+Relation type	+Novelty	Entity pair		+Relation type	+Novelty
JTIS	37.79	30.23	25.32		40.53	30.04	23.05
$JTI{S_{ensemble}}$	37.92	31.04	26.15		41.27	31.03	23.34
Median	–	–	–		24.15	17.76	13.31
Mean	–	–	–		26.46	19.66	14.91

### Ablation study

In order to investigate the effectiveness of each of the four complementary tasks proposed in “Methods” section, we conduct extensive ablation studies on the BioRED validation set by selectively omitting certain task modules during training.

As shown in Tables 4 and 5, without the use of any auxiliary tasks, the model’s performance is on par with the baseline provided in the original BioRED dataset paper, with any minor performance differences attributable to variations in data preprocessing. When employing a single auxiliary task, only the EPT is added, and the performance of entity pair extraction is the best, CR proves to be the most effective for recognizing the type of relationship after extraction, underscoring the importance of entity coreference information and entity pair type information in relation classification; for TNT, RE has the most substantial impact, highlighting the significance of the relationship category of entity pairs in determining if a triplet is a novel proposition within the text. When auxiliary tasks are paired, they enhance both RE and TNT performance. Notably, the combination of CR and TNT markedly enhances RE, demonstrating that integrating co-reference information with the semantic novelty of triplets significantly refines the semantic representation of entity pairs. The pairing of RE and EPT is the most effective dual-task configuration for TNT performance, effectively mitigating the issue of error propagation within the TNT task. The JTIS model, incorporating all auxiliary tasks, achieves the highest F1 scores in both RE and TNT tasks, demonstrating the distinct advantages of a full auxiliary task combination in promoting entity-relation mapping and enriched context representation.

**Table 4.** An ablation study conducted using our model on the triple extraction component to assess the effectiveness of three tasks (i.e. CR, EPT, and TNT) for RE based on the BioRED dataset (%)

CR	EPT	TNT	RE	Entity pair	+Relation type
			√	78.06	66.44
√			√	78.42	67.42
	√		√	78.91	66.82
		√	√	79.22	66.73
√	√		√	79.06	67.72
	√	√	√	79.75	67.62
√		√	√	79.67	68.02
√	√	√	√	79.82	68.10

**Table 5.** An ablation study conducted using our model on the relation novelty prediction component to assess the effectiveness of three tasks (i.e. CR, EPT, and RE) for the prediction of TNT based on the BioRED dataset (%)

CR	EPT	RE	TNT	+Novelty
			√	58.18
√			√	59.20
	√		√	58.42
		√	√	59.31
√	√		√	59.38
	√	√	√	59.53
√		√	√	59.82
√	√	√	√	60.13

### Case study

**Figure 4.**
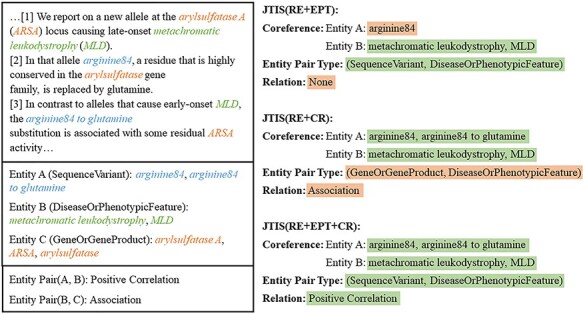
A case study of our method, showing a portion of entities and sentences, due to space constraints.

To gain a deeper understanding of how coreference information and entity type information assists in RE, we present a case study in Fig. 4. In this case, three different model configurations handle the task of identifying the relationship between entities, where Entity A and Entity B have a “Positive Correlation” relationship. Since TNT and RE are sequentially related, where TNT is designed to enhance the subsequent steps of RE and any error in RE inevitably leads to an error in TNT. Ablation experiments have already confirmed the effectiveness of using TNT loss to improve RE, so the following configurations all include the TNT module by default.

(i) JTIS (RE + EPT): This model combines RE with the entity type information task. During the process, the model makes an incorrect coreference judgment for Entity A, leading to a failure in extracting the correct relationship. (ii) JTIS (RE + CR): This model trains without entity classification loss, meaning it lacks the EPT module. The model incorrectly identifies the entity pair type, mistakenly interpreting the relationship between the entities as “Association.” These results suggest that relying solely on certain tasks may not sufficiently capture the complex relationships in the text. (iii) JTIS (RE + EPT + CR): This model integrates RE, EPT, and CR tasks. It correctly identifies the entity pair type and coreference relationship and accurately classifies the “Positive Correlation” relationship between Entity A and Entity B. This case study shows that integrating coreference and entity type information significantly enhances the model’s ability to identify entity relationships. Utilizing multiple tasks together helps the model more comprehensively understand and handle the complex relationships within the text.

Additionally, during output inspection, we find that the JTIS model still exhibits a considerable number of errors in classifying relationships between entity pairs that appear in different sentences. When entities are not expressed within the same sentence, the context becomes increasingly complex, making it harder for the model to find the correct relationship. In future work, we plan to incorporate anaphor information and optimize the loss calculation method to address class imbalance in the dataset. Moreover, our multistage entity linking process may add complexity to the system and potentially introduce errors between different components. Due to time constraints, we rely on the PubTator’s API to retrieve unlabeled entities. It can be observed that the model’s performance heavily depends on the accurate predictions of the Named Entity Recognition (NER) system. In the future, we plan to develop a more robust NER and entity normalization system to improve performance.

### Comparison with other approaches

In the BioCreative VIII challenge, many other teams also propose outstanding methods [32]. For example, one team employs a dual-classifier model using BioLinkBERT [33] for relation classification and novelty detection. They combine negative sampling with a multihead attention mechanism and improve accuracy through ensemble voting. However, this approach heavily relies on the choice of negative samples. To maintain dataset balance, the selection and number of negative samples significantly affect model performance, which adds complexity to model training. Another team enhances a BERT model using external domain knowledge in the form of ontologies, but this approach requires a large number of examples for labels with low representation, resulting in performance significantly lower than the task’s average and median levels. Some teams combine multiple specialized biomedical models (like BioBERT) with GPT-4 for data generation. However, this method is quite complex and may pose challenges in practical implementation. Additionally, another team proposes an Entity-Aware Masking strategy, which aims to enhance entity representation through a pre-trained masking strategy. Nevertheless, this method may require fine-tuning for specific tasks related to masked named entities, potentially limiting its applicability across different datasets.

In contrast, we propose a multitask joint learning approach, which addresses the issue of error propagation by enhancing intermediate steps in RE, thereby improving both relationship classification and end-to-end joint RE performance. Our key advantages lie in: (i) We uniquely decompose the intermediate steps of RE into multiple subtasks, and through joint learning, improve the model’s contextual understanding capabilities. Compared to other teams’ single-task learning approaches, joint learning allows the model to capture the connections between multiple tasks. (ii) We use PubMedBERT for contextual encoding and mark entities at their positions to better identify them, improving the model’s understanding of the semantic structure of the text. This strategy is particularly effective in DocRE, as it enables more precise identification of relevant entity pairs within the text. (iii) We enhance the model’s stability and generalizability through K-fold cross-validation and model ensemble techniques.

## Conclusion

In this paper, we propose explicitly training the model to capture coreference and entity type information related to the RE and TNT tasks through the joint training of multiple relevant tasks. By leveraging a carefully designed set of diverse tasks, our approach extracts relationships of higher quality due to more effective supervision, leading to improved accuracy for novel classification and alleviating the issue of error accumulation. Furthermore, we enhance the model’s performance through model ensemble. The experimental results demonstrate the effectiveness of our approach.

## Data Availability

The dataset used in this study is publicly available at https://codalab.lisn.upsaclay.fr/competitions/13377#learn_the_details-dataset.
